# Exploring the Nerve Regenerative Capacity of Compounds with Differing Affinity for PPARγ In Vitro and In Vivo

**DOI:** 10.3390/cells12010042

**Published:** 2022-12-22

**Authors:** Melissa L. D. Rayner, Simon C. Kellaway, Isabel Kingston, Owein Guillemot-Legris, Holly Gregory, Jess Healy, James B. Phillips

**Affiliations:** 1 Department of Pharmacology, School of Pharmacy, University College London, London WC1N 1AX, UK; 2 Centre for Nerve Engineering, University College London, London WC1N 6BT, UK

**Keywords:** peripheral nerve injury (PNI), peroxisome proliferator-activated receptor gamma (PPARγ), non-steroidal anti-inflammatory drugs (NSAIDs), nerve regeneration, functional recovery, drug affinity

## Abstract

Damage to peripheral nerves can cause debilitating consequences for patients such as lifelong pain and disability. At present, no drug treatments are routinely given in the clinic following a peripheral nerve injury (PNI) to improve regeneration and remyelination of damaged nerves. Appropriately targeted therapeutic agents have the potential to be used at different stages following nerve damage, e.g., to maintain Schwann cell viability, induce and sustain a repair phenotype to support axonal growth, or promote remyelination. The development of therapies to promote nerve regeneration is currently of high interest to researchers, however, translation to the clinic of drug therapies for PNI is still lacking. Studying the effect of PPARγ agonists for treatment of peripheral nerve injures has demonstrated significant benefits. Ibuprofen, a non-steroidal anti-inflammatory drug (NSAID), has reproducibly demonstrated benefits in vitro and in vivo, suggested to be due to its agonist action on PPARγ. Other NSAIDs have demonstrated differing levels of PPARγ activation based upon their affinity. Therefore, it was of interest to determine whether affinity for PPARγ of selected drugs corresponded to an increase in regeneration. A 3D co-culture in vitro model identified some correlation between these two properties. However, when the drug treatments were screened in vivo, in a crush injury model in a rat sciatic nerve, the same correlation was not apparent. Further differences were observed between capacity to increase axon number and improvement in functional recovery. Despite there not being a clear correlation between affinity and size of effect on regeneration, all selected PPARγ agonists improved regeneration, providing a panel of compounds that could be explored for use in the treatment of PNI.

## 1. Introduction

Even in the best-case scenario, with microsurgical therapy providing a supportive environment for regeneration, neuron growth rate is limited to ~1 mm/day [1]. Therefore, months to years can elapse before regenerating neurons reach their target organ. For motor neurons growing back to muscle, this delay leaves the muscle with no nerve stimulation over time, resulting in irreversible wasting. This means that even if the neurons eventually reach their target muscle, there is little recovery of function. The development of a drug that acts to accelerate nerve regeneration, with subsequent improvement in functional recovery, will provide a therapy for an unmet clinical need.

The role of peroxisome proliferator-activated receptor gamma (PPARγ) in nerve tissue has not been fully determined but there is evidence from rodent studies that it may be involved in neuronal development, neuronal health, and pain signalling [2]. Immunohistochemical analysis has demonstrated localisation of PPARγ in Schwann cells and endothelial cells in rat peripheral nerves [3], expression of PPARγ within Schwann cells of healthy and regenerating nerves following a crush injury [4], and an increase in expression during the inflammation process [5]. PPARγ expression has also been demonstrated in axons within a rat sciatic nerve at 2, 4, and 6 h after a nerve ligation or crush injury [6]. The presence and increased expression of PPARγ following a peripheral nerve injury (PNI) provide possible benefits for its use in developing therapies for this unmet clinical need.

PPARγ is an upstream mediator of the Rho/ROCK pathway [7] and its activation is suggested to inhibit this pathway via the upregulation of the protein tyrosine phosphatase, Src homology region 2–containing protein tyrosine phosphatase-2 (SHP-2). This cytosolic protein tyrosine phosphatase (PTP) dephosphorylates and inactivates guanine nucleotide exchange factor (GEF), Vav, which is turn suppresses the conversion of inactive guanosine diphosphate (GDP) -Rho to active guanosine triphosphate (GTP) -Rho [7]. As a consequence, ROCK is not activated and this inhibition of the Rho/ROCK pathway prevents stiffening of the actin cytoskeleton, encourages axonal elongation, and prevents growth cone collapse [8,9,10].

PPARγ is one of several upstream effectors of the Rho/ROCK pathway [11], making it a great target for drug therapies. Pharmacological treatment of PNI with PPARγ agonists such as non-steroidal anti-inflammatory drugs (NSAIDs) and **thiazolidinediones** has shown beneficial effects on nerve regeneration, with the opposite effect seen with the PPARγ antagonist, GW9662 [6]. Thiazolidinediones are antidiabetic agents that are potent and selective activators of PPARγ [12]. Their use in PNI has not been extensively explored but increased neurite outgrowth was seen following troglitazone-treated primary rat hippocampal cultures [13] and rosiglitazone-treated primary rat cortical neurons [6].

Ibuprofen and diclofenac are the most commonly studied NSAIDs for PNI treatment [9,14,15,16]. The pharmacological effects of NSAIDs are thought to be independent of their well-known anti-inflammatory activity on cyclooxygenase (COX) 1 and 2 [17,18]. The characteristic lipophilic backbone and acid moiety, most commonly a carboxylate, within the structure of NSAIDs is what may enable them to bind to PPARγ [19]. There are several classes of NSAIDs based upon their chemical structure including thiazinecarboxamides (piroxicam), derivatives of arylacetic acid (indomethacin), aminoarylcarboxylic acid (flufenamic acid), arylpropionic acid (ibuprofen and fenoprofen), and salicylic acid (aspirin) [19]. The different classes of NSAIDs have demonstrated a spectrum of agonist activity on PPARγ. Ibuprofen, fenoprofen, and flufenamic acid exhibit the greatest effect on PPARγ with indomethacin, piroxicam, and salicylic acid having little or no effect [19]. This correlated with studies identifying reduced levels of activated Rho with ibuprofen, indomethacin, and sulindac sulfide treatment, but not with piroxicam, meloxicam, and naproxen [18,20,21,22]. The reduced levels of activated Rho correlated to an increase in axon growth when treated with ibuprofen and indomethacin but not with naproxen [18,20,21].

Previous literature has identified and discussed the relative affinities of NSAIDs for PPARγ (sulindac sulfide > diclofenac > indomethacin > ibuprofen) [23,24]. Given the promising results already seen with ibuprofen and diclofenac, other NSAIDs could have promising effects on nerve regeneration and may even exceed those already seen. In contrast, naproxen has been shown not to activate PPARγ in neuronal cells [20], and not to have a RhoA-suppressing function [25].

Gaining an understanding of how common compounds such as **thiazolidinediones and NSAIDs** interact with PPARγ could aid the development of more potent and selective agonists, corresponding to a greater effect on nerve regeneration and functional recovery following a PNI. Therefore, the aims of this study were to investigate the regenerative capacity of a panel of PPARγ agonists and determine whether there is a correlation between a compound’s affinity for PPARγ and its capacity to enhance neuronal regeneration in vitro and in vivo.

## 2. Materials and Methods

### 2.1. Cell Cultures

#### 2.1.1. SCL4.1/F7 Schwann Cell Line

Cells from the rat Schwann cell line SCL4.1/F7 (Health Protection Agency) were grown in Dulbecco’s Modified Eagle Medium (DMEM) high glucose medium supplemented with 10% *v*/*v* heat inactivated fetal bovine serum (FBS) and 100 U/mL of Penicillin, 100 µg/mL of Streptomycin, in standard cell culture flasks. The cultures were maintained at sub-confluency at 37 °C with 5% CO_2_ and passaged when required.

#### 2.1.2. PC12 Neuronal Cell Line

PC12 cells (Pheochromocytoma cells from rat adrenal medulla used as a neuronal cell line, 88022401; Sigma-Aldrich, St. Louis, MO, USA) were grown in suspension in RPMI 1640 medium supplemented with 100 U/mL of Penicillin, 100 µg/mL of Streptomycin, 2 mM L-glutamine, 10% *v*/*v* heat-inactivated horse serum and 5% *v*/*v* FBS in standard cell culture flasks. The cultures were maintained at sub-confluency at 37 °C with 5% CO_2_ and passaged when required.

### 2.2. Fabrication of 3D EngNT Co-Cultures

Anisotropic cellular gels were prepared as described previously [26]. Briefly, 1 mL of solution containing 80% *v*/*v* type I rat tail collagen (2 mg/mL in 0.6% acetic acid), 10% *v*/*v* minimum essential medium, 5.8% *v*/*v* neutralising solution, and 4.2% Schwann cell suspension (4 × 10^6^ SCL4.1/F7 cells per 1 mL gel) was integrated with tethering mesh at opposite ends of a rectangular mold (Dimensions 16.4 mm × 6.5 mm × 5 mm) [27]. Cellular gels were immersed in 10 mL DMEM and incubated at 37 °C with 5% CO_2_ for 24 h to enable cellular self-alignment, then stabilised using plastic compression.

Plastic compression was conducted by transferring the gel to a standard blotting element, which comprised of a layer of stainless-steel support mesh covered by a layer of nylon mesh placed on top of three layers of circular Grade 1 Whatman filter paper [26]. The gel was then covered with a second layer of nylon mesh and a glass sheet. Compression was then completed by placing a 120 g stainless steel weight on top of the blotting element (stress equivalent to approximately 1.8 kN/m^2^) for 1 min.

Each stabilised aligned cellular gel was cut into 4 equal segments to obtain a control and three test regions from each gel. Each gel segment was transferred to a separate well in a 24-well plate, then 100,000 PC12 cells were seeded on top of each segment for co-cultures. The gels were incubated for 1 h at 37 °C to allow attachment of neuronal cells to the collagen gel, then 1 mL of culture medium (DMEM high glucose), supplemented with 10% heat inactivated FBS and 100 U/mL of Penicillin and 100 µg/mL of Streptomycin, was added to each well. The neurons were seeded onto the top surface of the gels and neurites extended across the horizontal plane following the aligned Schwann cells.

Three-dimensional EngNT co-cultures containing SCL4.1/F7 and PC12 cells, as described previously [28], were subjected to drug treatments for 72 h before being fixed with 4% *w*/*v* paraformaldehyde (PFA) in PBS at 4 °C overnight for subsequent immunostaining and microscopy analysis. Drug stock solutions made up in DMSO were added directly to the media in the appropriate volume to provide the required drug concentration.

### 2.3. Immunocytochemistry

Following fixation with 4% (*w*/*v*) PFA in PBS gels were washed 3 times with PBS and permeabilised using 0.5% (*v*/*v*) Triton-X-100 in PBS. Washes were repeated before blocking (goat serum 1/20 in PBS). Gels were washed before the addition of anti-mouse βIII –Tubulin primary antibody (Sigma-Aldrich T8660) diluted in PBS and incubated overnight at 4 °C. Washes were repeated before adding the corresponding secondary antibody (Dylight anti-mouse IgG 488 (1:400, diluted in PBS) (Thermofisher 35502, Paisley, UK). The secondary antibody was washed, and the sample was stored at 4 °C before viewing. Omission of primary or secondary antibody was routinely used as a control. When required, Hoechst was used to stain nuclei by incubating the gel for 15 min and then washed thrice with PBS.

### 2.4. Surgical Nerve Injury Models In Vivo

All surgical procedures were performed in accordance with the UK Animals (Scientific Procedures) Act (1986), the European Communities Council Directives (86/609/EEC) and approved by the UCL Animal Welfare and Ethics Review Board (AWERB). Adult male Wister rats (250–300 g) (Charles River) were randomised into groups and housed in plastic cages with soft bedding and free access to food and water. The animals were deeply anaesthetised by inhalation of isoflurane, and the left sciatic nerve was exposed at mid-thigh level. This was done by making an incision (~3 cm) parallel to the femur between the knee and hip then separating muscle layers to expose the nerve. Under the microscope (Zeiss CL 1500 ECO, Carl Zeiss GmbH), the sciatic nerve was released from the surrounding tissue.

A crush injury (axonotmesis) was achieved by applying a consistent pressure with a pair of sterile TAAB type 4 tweezers closed fully on the same point of the nerve (1.5 cm distal of the femur) for 15 s. This was repeated twice more in the same location with the tweezers positioned perpendicular to the nerve and rotated through 45° between each crush application. A 10/0 epineurial suture (Ethicon) was used to mark the injury site. Following the injury an osmotic pump (Alzet, model 1004, Cupertino, CA, USA) loaded with 100 µL of drug solution in PBS was implanted locally parallel to the sciatic nerve, delivering drug at a rate of 0.11 µL/h. The overlying muscle layers were closed using two 4/0 sutures (Ethicon, W2850, London, UK) and the skin was closed using stainless steel wound clips. The animals were allowed to recover and were maintained for 28 days.

All animals received the same level of interaction throughout the study. They were handled prior to surgery and repeatedly throughout the study for training and completion of functional testing. All animals were left to settle (~5 min) before conducting any functional tests.

### 2.5. Nerve Tissue Harvest

Animals were culled using an overdose of anesthetic according to local regulations, and the repaired nerves (~1.5 cm) were excised under an operating microscope and cut as required for analysis. The nerve tissue was immersion-fixed in 4% (*w*/*v*) PFA in PBS at 4 °C overnight.

### 2.6. Nerve Tissue Analysis

#### 2.6.1. Cryo-Sectioning

Following fixation, the nerve samples were incubated in 30% sucrose overnight and underwent subsequent snap freezing in 1:1 FSC 22 Frozen Section Media (Leica) and 30% sucrose. Transverse sections (10 μm thick) were prepared from the distal stumps, at a defined distance from the injury site, using a cryostat (Fisher scientific HM525 NX, Loughborough, UK). The sections were adhered to glass slides (Superfrost™ Plus) for histological analysis.

#### 2.6.2. Immunohistochemistry

Nerve sections were washed in immunostaining buffer (PBS together with 0.2% Triton-X), 0.002% sodium azide, and 0.25% Bovine Serum Albumin before the addition of serum to block non-specific binding (goat serum 1/20 in immunostaining buffer) for 45 min. The blocking serum was removed, and sections were incubated with anti-mouse neurofilament-H primary antibody diluted in immunostaining buffer overnight at 4 °C. The sections were washed with immunostaining buffer before addition of the secondary antibody and incubation at room temperature for 45 min. Sections underwent a final wash with immunostaining buffer before mounting with Vectashield Hardset mounting medium with DAPI.

### 2.7. Image Analysis and Quantification

Fluorescence microscopy (Zeiss Axiolab A1, Axiocam Cm1, Carl Zeiss GmbH) was used to capture images of neurites from five pre-determined fields of each gel using a ×20 lens. The positions of the pre-determined fields on the gels were equally spaced (625 μm apart) in a line along the edge of the construct where alignment was greatest [27]. The length of each neurite in each field (~1–12 neurites) was measured using Fiji ImageJ [29]. Following stabilization, the gel acquired a thickness of 100 μm and the neurons extended predominantly in a single horizontal plane along the top surface, following the aligned Schwann cells [30].

Tile scans were used to capture high-magnification (×20) micrographs from the entire nerve cross-section using a Zeiss LSM 710 confocal microscope and images were analysed using Volocity™ 6.4 (PerkinElmer, Beaconsfield, UK) running automated image analysis protocols to determine the number of neurofilament-immunoreactive neurites in each transverse nerve section.

### 2.8. Functional Outcomes In Vivo

#### 2.8.1. Electrophysiology

After 28 days, animals were anaesthetised using isoflurane and nerve function was assessed by electrophysiology (Synergy Ultrapro 3) by comparing the repaired nerve to the contralateral undamaged nerve in each animal. Monopolar needle electrodes were attached to the animal; a grounding electrode was placed in the tail of the animal and a reference electrode was placed above the hip bone. A stimulating electrode was placed against the proximal nerve 2 mm above the injury site and a recording needle was placed into the gastrocnemius muscle. The distance between the stimulating and recording electrodes was standardised. The nerve was stimulated using a bipolar stimulation constant voltage configuration and the muscle response recorded. The stimulation threshold was determined by increasing the stimulus amplitude in 0.1 V steps (200 μs pulse), until both a supra-maximal muscle action potential was recorded, and a significant twitch of the animal’s hind paw was seen. The amplitude (mV) of the compound muscle action potential (CMAP) was measured from the baseline to the peak, and the latency was measured from the time of stimulus to the first deviation from the baseline. Muscle action potential measurements were conducted in triplicate for both the injured nerve and contralateral control nerve in each animal.

#### 2.8.2. Static Sciatic Index (SSI)

Functional recovery was analysed using the static sciatic index (SSI). The animal’s hind paws were imaged from below with the animal standing on a Perspex platform and the toe spread factor (TSF), between the 1st and 5th toe, and the intermediary toe spread factor (ITSF), between the 2nd and 4th toe, were measured and Equation (1) was used to calculate SSI [31].

SSI = (108.44 × TSF) + (31.85 × ITSF) − 5.49

TSF = (TS_injury_ − TS_control_)/TS_control_ (1)

ITSF = (ITS_injury_ − ITS_control_)/ITS_control_

#### 2.8.3. Von Frey

The animals were placed on a grid and von Frey filaments made of nylon, which all have the same length but vary in diameter to provide a range of forces (0.008–300 g), were applied through the underside of the grid to stimulate the centre of the animal’s hind paws. A response was measured by the retraction of the animal’s paw in response to the filament stimulus. The threshold response was recorded by decreasing the stimulus until no response was detected.

### 2.9. Statistical Analysis

Normality tests were conducted on all data to determine appropriate statistical tests, and a one-way analysis of variance (ANOVA) was performed, as data followed a normal distribution. A one-way ANOVA was followed by a Dunnett’s post hoc test. For all tests, ^∗^ *p* < 0.05, ^∗∗^ *p* < 0.01, ^∗∗∗^ *p* < 0.001 and ^∗∗∗∗^ *p* < 0.0001 were considered significant.

## 3. Results

### 3.1. Regenerative Capacity of PPARγ Agonists In Vitro

**Figure 1:**
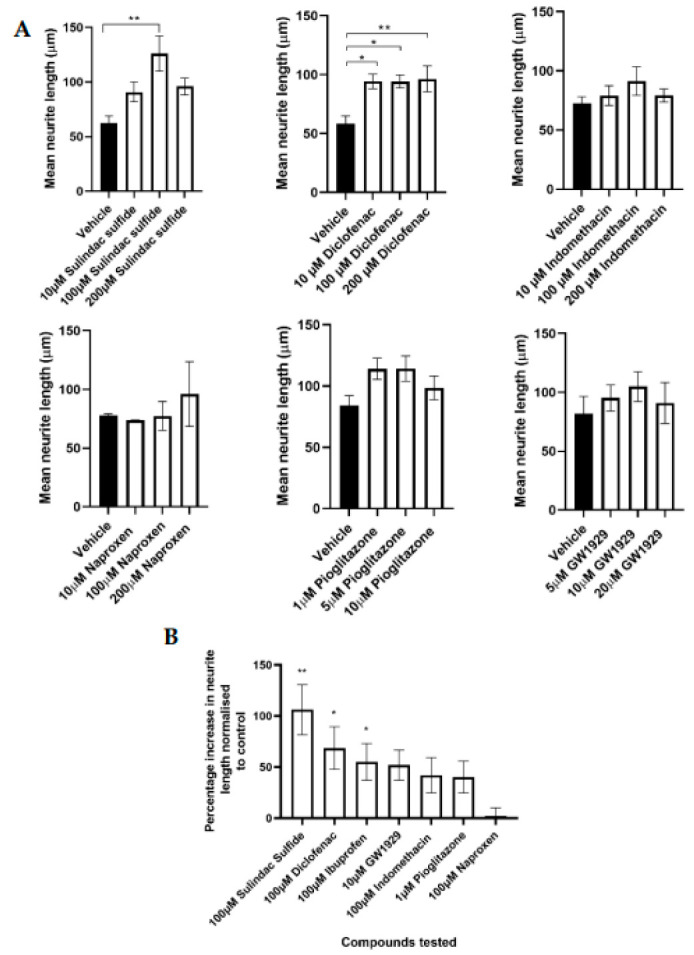
PC12 neuronal cells were seeded upon EngNT containing SCL4.1/F7 Schwann cells. (**A**) Significant increases in neurite length were seen in the presence of 10 µM, 100 µM, and 200 µM diclofenac and 100 µM sulindac sulfide compared to the vehicle control, after 72 h exposure. (**B**) The percentage increase in neurite length normalised to the control showed that sulindac sulfide had the greatest effect on neurite outgrowth and naproxen the least. Scale bar = 100 μm. N = 6, mean ± SEM for each condition. One-way ANOVA with Dunnett’s post hoc test, * *p* < 0.05 and ** *p* < 0.01.

PC12 neuronal cells seeded upon EngNT containing SCL4.1/F7 cells were treated with compounds sulindac sulfide, diclofenac, indomethacin, naproxen, pioglitazone, and GW1929 to modulate neurite outgrowth. Dose responses were conducted for each compound to determine their therapeutic window (Figure 1A). Doses tested in vitro were selected from previous literature to ensure non-toxic doses were used. The doses of the NSAIDs were kept the same as those previously used with ibuprofen [28] to make the results comparable across the drug class (some of these drugs had not been tested in PNI models previously and so no literature was available). Treatments with 10 µM, 100 µM, and 200 µM diclofenac and 100 µM sulindac sulfide significantly increased neurite extensions in comparison to the vehicle control (Figure 1A). Ibuprofen had previously shown to increase neurite growth and GW9662 to have no effect in the same model [28].

Table 1 shows the neurite length as a percentage increase to the experimental control, measured from co-cultures containing Schwann cells (SCL4.1/F7) and neurons (PC12s). Neurite length following the treatment with ibuprofen and GW9662 has been published previously [28]; the effect of the other compounds is presented in this study.

Comparing a dose of 100 µM of each of the five NSAIDs and the optimal dose of pioglitazone (1 µM) and GW1929 (10 µM) indicated some correlation between the agonist’s affinity for the receptor and increases in neurite outgrowth (Table 1).

Sulindac sulfide had the greatest effect on nerve regeneration, with a 106% increase in neurite length relative to the vehicle control (Figure 1B) and naproxen, which has been found not to activate PPARγ, showing minimal effect on nerve regeneration (Table 1).

### 3.2. Effect on Neuronal Growth of PPARγ Agonists In Vivo

**Figure 2:**
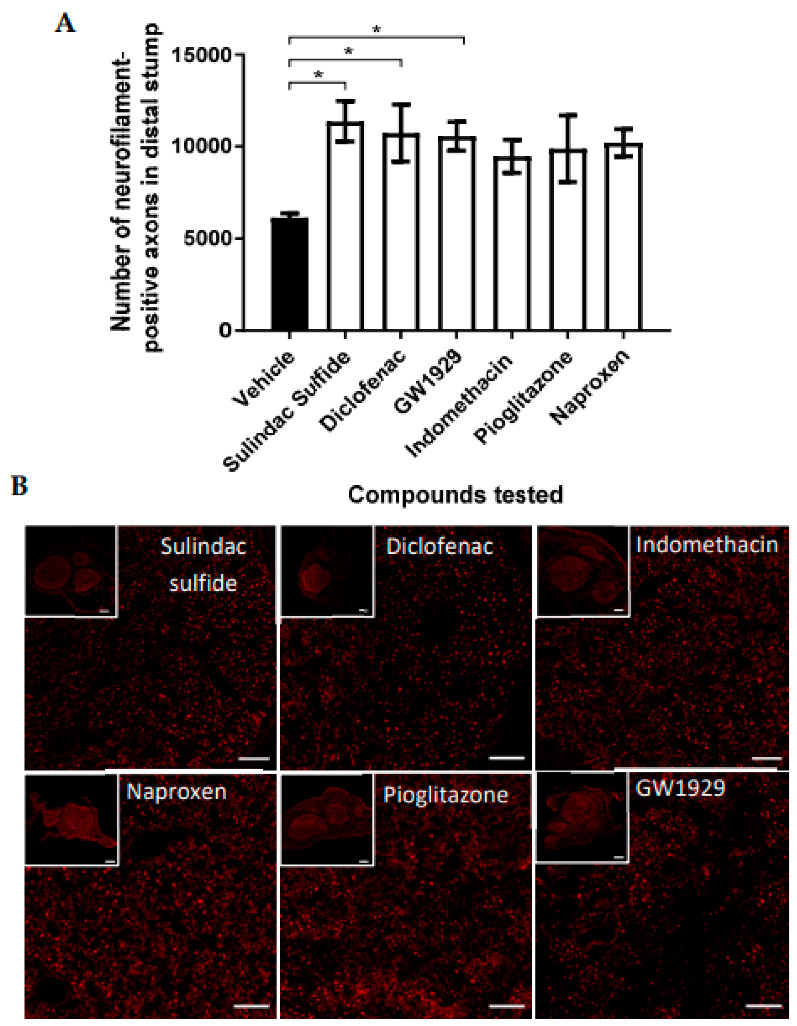
(**A**) The number of neurofilament-positive axons in the distal stump appeared to be elevated with all PPARγ agonists in comparison with the vehicle control, with statistical significance seen with sulindac sulfide, diclofenac, and GW1929 local treatment. Doses delivered locally to the site of injury were 11 µg/day diclofenac, 11 µg/day sulindac sulfide, 2 µg/day GW1929, 12 µg/day indomethacin, 0.12 µg/day pioglitazone, and 8 µg/day naproxen. (**B**) Micrographs are transverse sections showing neurofilament-positive neurites at 5 mm distal to the injury site. Scale bar = 100 μm. N = 6, means ±  SEM. One-way ANOVA with Dunnett’s post hoc test, * *p* < 0.05.

Agonists were released in a controlled manner locally at the nerve injury site using Alzet osmotic pumps. The doses delivered were based upon the dose given in vitro. Neurofilament-positive axons were quantified from 10 µm transverse rat sciatic nerve sections taken 5 mm distal to the injury site at 28 days post injury. All agonists increased the number of neurites present in comparison to the vehicle, however, statistically significant differences were only seen with 11 µg/day diclofenac, 11 µg/day sulindac sulfide, and 2 µg/day GW1929 treatment and not 12 µg/day indomethacin, 0.12 µg/day pioglitazone, and 8 µg/day naproxen (Figure 2).

### 3.3. Functional Recovery Following In Vivo Treatment with PPARγ Agonists

**Figure 3:**
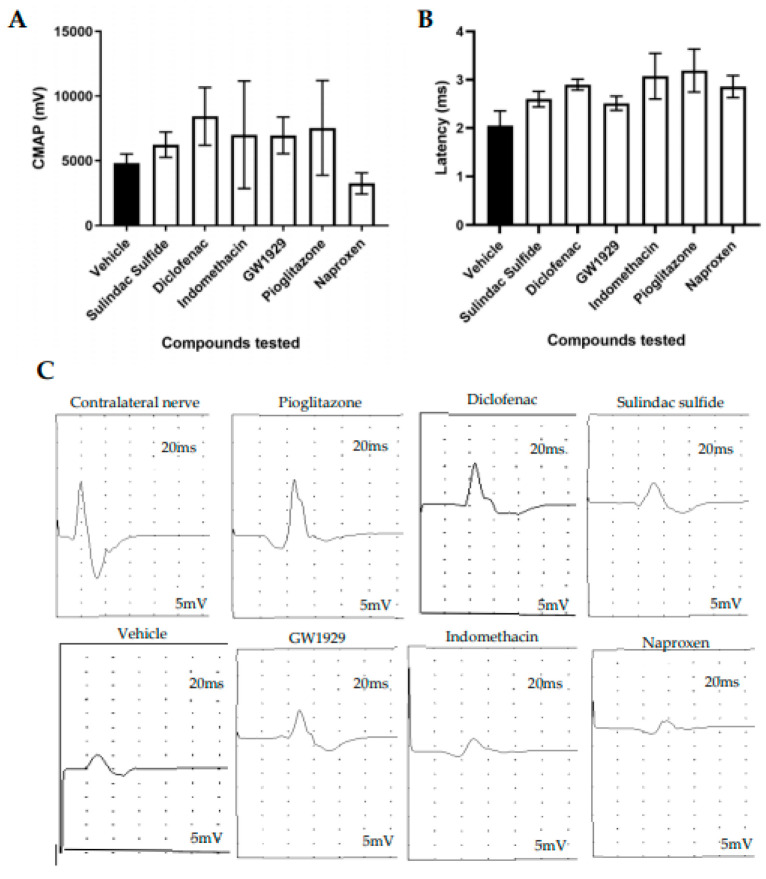
The sciatic nerve was stimulated proximal to the repair site and the CMAP was recorded from the gastrocnemius muscle. The CMAP amplitude reduced in the injury side in comparison with the contralateral non-injured side. (**A**) All compounds tested showed a trend towards a higher CMAP in comparison to the vehicle except naproxen, but no statistical significance was seen. (**B**) All compounds tested showed a trend towards a higher latency in comparison to the vehicle, but no statistical significance was seen. (**C**) The electrophysiological traces for the contralateral uninjured nerve and injured nerves. N = 6, means ± SEM.

To determine motor functional recovery, electrophysiology was used to measure the compound muscle action potential (CMAP) from the gastrocnemius muscle during electrical stimulation of the proximal nerve. CMAP amplitude and latency recordings from the contralateral nerves were consistent between animals (data not shown). Variability was seen between animals, with a trend that all PPARγ agonists increased the CMAP, except naproxen, in comparison to the vehicle group but with no statistical significance (Figure 3A). All compounds tested showed a trend towards a higher latency in comparison to no drug treatment, but no statistical significance was seen (Figure 3B).

**Figure 4:**
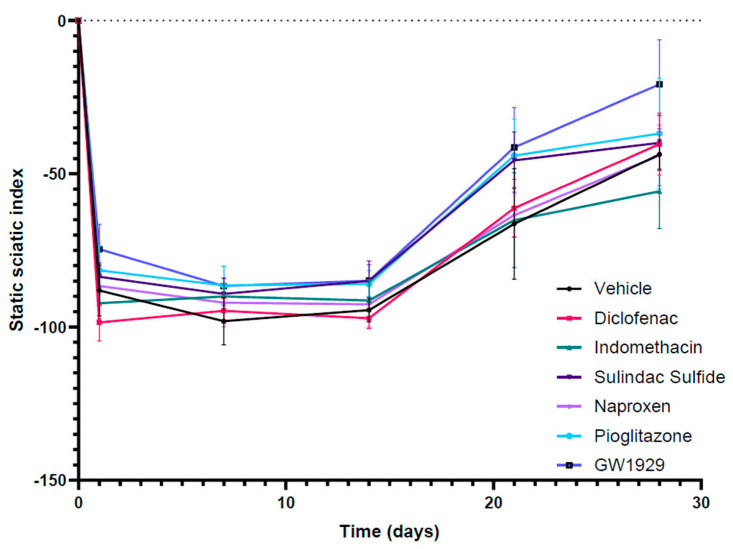
Hind paw images were used to conduct SSI quantitation in contralateral uninjured and injured sciatic nerves weekly until the 28-day endpoint. An initial loss of function can be seen immediately after the crush injury with recovery seen in all groups returning towards the baseline by 28 days post injury. N = 6, means ± SEM.

To further monitor motor function, static sciatic index (SSI) was measured weekly post injury alongside von Frey to monitor sensory recovery. In both outcomes, recordings demonstrated an initial loss of function immediately after the crush injury with recovery seen in all groups returning towards the baseline by 28 days post injury. No difference was seen between the groups following SSI (Figure 4) and von Frey. 

## 4. Discussion

Increased knowledge of the signaling pathways activated following a PNI, particularly the Rho/ROCK pathway, has helped the development of target-specific treatments with improved regeneration [35]. The inhibition of the Rho/ROCK pathway has been linked with the activation of PPARγ via the upregulation of the SHP-2, providing a platform for pharmacological intervention [7,20]. This study selected a panel of PPARγ agonists which were found to have a beneficial effect on nerve regeneration in vitro and in vivo. Additionally, it was found that there was some correlation between a drug’s affinity for PPARγ and its capacity to promote neurite outgrowth in vitro, however, this wasn’t apparent in vivo using a nerve crush model.

Using a 3D co-culture in vitro model containing neurons and Schwann cells and a sciatic nerve crush injury model in animals, we investigated the pro-regenerative effects of diclofenac, sulindac sulfide, indomethacin, naproxen, piogliazone, and GW1929. Ibuprofen has been tested in the same models in our previous studies [28]. Co-cultures comprised of both neuronal cells and Schwann cells were used to best mimic the native nerve environment following injury. There has been evidence of PPARγ activity in both neurons and Schwann cells [3,4,5,6], however, this was not explored in this study.

Ibuprofen is the most commonly studied NSAID in PNI promoting neurite growth in primary cell cultures in a dose-dependent manner [18,20,21]. In our previous study we presented a 55.2% increase in neurite growth following ibuprofen treatment compared to the vehicle in vitro. Further studies demonstrated that ibuprofen successfully increased axon number and functional recovery in animal studies [9].

Sulindac is a NSAID indicated for the relief of signs and symptoms of arthritic conditions, including osteoarthritis and rheumatoid arthritis. Sulindac is a pro-drug that undergoes reversible reduction to two metabolites: sulindac sulfide, which is of interest to us as it inhibits COX and Ras-mediated signal transduction, and sulindac sulfone [36,37]. Sulindac sulfide demonstrated the greatest effect on the neurite outgrowth in the 3D co-culture model by increasing the growth by 106% and demonstrated the highest axon number in the distal stump following the in vivo study. The drug’s effect on neurite growth has not been reported before, however sulindac sulfide has previously shown to inhibit the activity of Rho in a concentration-dependent manner. The direct effect of sulindac sulfide on Rho activation was explored in SY5YAPPswe, HEK 293, and PC12 cells and the levels of active Rho-GTP were found to be reduced in all the cell lines [22]. This suggests that sulindac sulfide has a similar mechanism of action to the other NSAID by its activation of PPARγ linking to the inhibition of the Rho/ROCK pathway.

Diclofenac is another widely used NSAID as an analgesic and anti-inflammatory. In nerve regeneration, diclofenac has been found to have conflicting effects by both improving regeneration but also having harmful effects on developing nerves [38,39,40,41]. Diclofenac has been shown to be teratogenic; during the embryonic period, the number of nerve fibers and the cross-sectional area of axons in developing sciatic nerves were affected when exposed to diclofenac [39]. Prenatal administration of the drug impairs the sciatic, optic, and median nerve development in the child. Furthermore, diclofenac has been found to inhibit neuronal stem cell differentiation and reduce proliferation through the apoptotic pathway [42].

In comparison, this study demonstrated that diclofenac significantly promoted nerve regeneration in both a 3D co-culture in vitro model and crush injury animal model. This echoed another study’s work that identified beneficial effects with diclofenac treatment in a transection injury on the rat sciatic nerve; by 16 weeks, increased regeneration was seen with diclofenac treatment suggested by a significant increase in gastrocnemius muscle weight, improved nerve fiber number, and axon diameter [14].

Indomethacin also increased neurite growth (19.3%) in the 3D co-culture model, however the effect was not as great as that seen with diclofenac (73%), ibuprofen (55.2%), or sulindac sulfide (106%). This mirrored the stimulation of neurite growth seen in a dorsal root ganglion (DRG) neuron culture when treated with indomethacin following exposure to inhibitory substrates [21]. Furthermore, the same study showed that indomethacin and ibuprofen blocked lysophosphatidic acid-induced Rho A activation in PC12 and DRG neuronal cells [21]. Indomethacin increased axon number in vivo but this was not significant.

**Figure 5:**
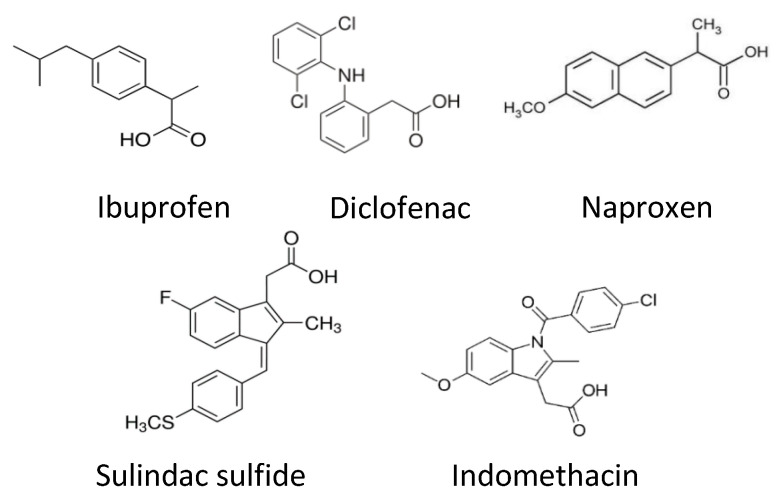
Chemical structures of NSAIDs.

The final NSAID tested was naproxen which had no effect on neurite growth in vitro. The different regenerative capacities between the five NSAIDs tested may be a result of their different chemical structures (Figure 5). This hypothesis has not been explored in this study, but it has been suggested that the PPARγ binding site cannot accommodate the CCH_3_ group on the structure of naproxen [25].

Two selective PPARγ agonists were also tested in this study: pioglitazone and GW1929. GW1929 is a research compound and was used as a positive control. Pioglitazone is an anti-diabetic drug [43] that has previously demonstrated benefit following nervous system injury by providing neuroprotection succeeding focal cerebral ischemia in rats [44], and promoting remyelination following a sciatic nerve crush injury in mice [45]. In the 3D co-culture, pioglitazone stimulated an increase in neurite growth (40%) even at a lower dose than the other agonists.

Regeneration and functional recovery were measured 28 days post injury after the compounds were delivered locally to the injury site in a controlled manner using Alzet osmotic pumps. Histological analysis demonstrated a trend in the increase in axon number in the distal stump with indomethacin, pioglitazone, and naproxen and significant increases seen with diclofenac, sulindac sulfide, and GW1929. The extent to which the agonists improved axon number differed in vivo to what we observed in vitro. The greatest difference was seen with naproxen treatment; no regeneration was observed in vitro, but the compound increased the axon number in the distal stump in comparison to the vehicle in vivo. [21]) found that the effects of ibuprofen via the inhibition of the Rho A pathway are independent of COX action. However, for naproxen to have an effect it must be either through its inhibition of COX or another unknown mechanism. This could suggest that the NSAIDs are acting through a dual mechanism in PNI which needs to be explored further.

The electrophysiological results demonstrated a trend towards an increase in CMAP with all PPARγ agonists except naproxen, however none were significant. This suggests that there may have been an improvement in target muscle reinnervation with all treatments except naproxen. No significance was seen in latency in any of the treatment groups in comparison to the vehicle group. In all groups, the SSI and von Frey recordings returned to baseline in a similar manner over the 28-day experiment; however, no differences were seen between the groups.

The list of NSAIDs that have a demonstrated effect in PNI are not limited to the ones tested in this study. Celecoxib, a COX-2 inhibitor, was tested in a sciatic nerve crush injury in rats, which resulted in improved functional recovery. Significant improvements were observed in the sciatic functional index (SFI), which was considered to be a result of neuroprotection [46]. In the same model in mice, acetylsalicylic acid also improved functional recovery seen in the SFI, nociception, and gait [47]. In a transection injury model in a sciatic nerve being fixed to the adjacent muscle, ketoprofen improved functional recovery and enhanced regeneration of axons [48]. Exploring how other NSAIDs promote nerve regeneration could help us decipher the mechanisms through which these agonists are working and aid the development of a successful drug therapy for PNI.

## 5. Conclusions

In conclusion, our results indicate that the relationship between affinity of the selected agonists for PPARγ and their capacity to promote nerve regeneration is complex as they could be working through additional mechanisms. There is previous evidence of PPARγ activity in the different neural cell types—both neurons and Schwann cells [3,4,5,6]. It has also been reported that PPARγ has a role in the activation of macrophages [49], with a further study demonstrating a correlation between PPARγ activation and M2 macrophage (regenerative phenotype) marker expression [50]. Additionally, a study has explored the partial and full agonistic activity and binding properties of these compounds on PPARγ [23], which could explain the differences seen in their regenerative capacity. Further in vivo studies using various injury models over longer time periods would help us decipher these mechanisms and which cell types these compounds are working on. Additional outcome measures could be completed to broaden the understanding of how these drugs are benefiting functional recovery. Target innovation could be evaluated using, for example the foot flick test or motor unit number estimation (MUNE) and functional recovery using a rotarod test, grasping test, or algesimetry test [51].

Furthermore, additional external factors that impact nerve regeneration could be evaluated alongside drug treatments such as exercise and rehabilitation. Physical activity has demonstrated to be beneficial in many pathologies due to improvement in health and well-being and has been found to have a positive effect on axonal growth, phenotypic changes in structures, and neurotrophin levels following PNI [52].

Finally, the work presented here supports the view that PPARγ is a suitable target for drug therapies for PNI and further development in this area could prove to be promising in translating drug therapies for PNI into the clinic. Repositioning current approved drugs such as the NSAIDs could achieve this more rapidly as their safety profiles required for regulatory approval are already established.

## Figures and Tables

**Table 1:** The ranking order of an agonist’s EC_50_ for PPARγ and neurite length increase in vitro.

Compound	PPARγ EC_50_	Dose of Compound Tested In Vitro (µM)	Neurite Length Seen with Optimal Dose Percentage Increase to the Control (Mean ± SEM)
**GW1929**	13 nM [32]	10	51% ± 14.74
**Pioglitazone**	380 nM [33]	1	40% ± 15.58
**Sulindac** **sulfide**	1.87 µM [23]	100	106% ± 24.61
**Diclofenac**	1 µM [23]	100	73.93% ± 28.65
**Ibuprofen**	56.8 µM [23]	100	55.2% ± 17.92 [28]
**Indomethacin**	21 µM [23]	100	19.3% ± 17.35
**Naproxen**	No action [20]	100	1.8% ± 8.44
**GW9662**	2 nM [34] Irreversible Antagonist	100	−89% ± 10.41 [28]
